# Immunomodulatory and growth-promoting effects of *Rauwolfia serpentina* root powder in broiler chicks challenged with *Salmonella* Gallinarum

**DOI:** 10.3389/fvets.2025.1534347

**Published:** 2025-02-03

**Authors:** Yingyu Zhang, Hiba Rehman, Farina Khattak, Maryam Tariq, Bushra Nisar Khan, Sadia Chaman, Ayaesha Riaz, Muhammad Ovais Omer, Aqib Ali, Qamar un Nisa, Muhammad Muddassir Ali, Gulbeena Saleem

**Affiliations:** ^1^Henan Key Laboratory of Rare Diseases, Endocrinology and Metabolism Center, The First Affiliated Hospital, and College of Clinical Medicine of Henan University of Science and Technology, Luoyang, China; ^2^Department of Pathology, University of Veterinary and Animal Sciences, Lahore, Pakistan; ^3^Monogastric Science Research Centre, Scotland's Rural College (SRUC), Edinburgh, United Kingdom; ^4^Institute of Zoology, University of Punjab, Lahore, Pakistan; ^5^Institute of Pharmaceutical Sciences, University of Veterinary and Animal Sciences, Lahore, Pakistan; ^6^Department of Parasitology and Microbiology, Faculty of Veterinary and Animal PMAS-ARID Agriculture University, Rawalpindi, Pakistan; ^7^Department of Pharmacology and Toxicology, University of Veterinary and Animal Sciences, Lahore, Pakistan; ^8^Institute of Biochemistry and Biotechnology, University of Veterinary Animal Sciences, Lahore, Pakistan

**Keywords:** *Rauwolfia serpentina*, *Salmonella* Gallinarum, immunity, gene expression, gut morphology, broiler chicks

## Abstract

**Background:**

Amid growing concern about antimicrobial resistance due to the irrational use of antibiotics in treating common poultry diseases, particularly *Salmonella* which is a foodborne pathogen in humans. This study investigates the effects of ethnoveterinary supplementation of *Rauwolfia serpentina* (L. Benth. ex Kurz) powder (RSP) on three key immune-related genes; Suppressor of cytokine signaling 3 (SOCS3), the quiescence-related gene P20K (P20K), and the major histocompatibility complex Class IIβ (MHC class IIβ), gut morphology and growth performance of broiler chicks infected with *Salmonella* Gallinarum.

**Methods:**

Two hundred and forty day-old Hubbard classic chickens were randomly assigned to four groups: non-challenged control (NC), and *Salmonella* Gallinarum challenge group (SGC), and two treatment groups fed a basic diet supplemented with 1.5% *Rauwolfia serpentina* powder (RSP) with SGC (RSP-1) and 3% RSP with SGC (RSP-2), respectively, from day 3 till 28 days of age. Each treatment was replicated 4 times with 15 bird/replicate pen. On day 7, all the birds in the RSP-1, RSP-2 and SGC groups received 1 ml of BHI broth containing 2 × 108 CFU of *Salmonella* Gallinarum via oral gavage. While control birds received an equivalent volume of sterile BHI broth. Gene expression analysis was conducted using real-time PCR to measure the expression of key immune-related genes: SOCS3, P20K, and MHC Class IIβ in spleen, liver, and caeca. Additionally, histopathological assessments of gut and growth performance parameters including feed intake, body weight gain, and feed conversion ratio (FCR) were monitored throughout the experimental period.

**Result:**

The gene expression analysis at 3 and 21 days post-challenge revealed that SGC birds had significantly higher SOCS3, P20K, and lower MHC class IIβ expression (*p* < 0.001) in the caecum, liver, and spleen of broiler chickens. In contrast, the RSP-1 and RSP-2 groups showed significantly lower SOCS3 and P20K expression (*p* < 0.001), alongside improved gut morphology, weight gain, and FCR compared to the SGC group, with these benefits increasing over time.

**Conclusion:**

In conclusion, these findings suggest that *Rauwolfia serpentina* supplementation modulates key immune-related gene expression (SOCS3, P20K, and MHC class IIβ), enhances intestinal health, and improves growth performance in broilers challenged with *Salmonella* Gallinarum.

## 1 Introduction

*Salmonella enterica* subspecies *enterica* serovar Gallinarum biotype Gallinarum (*Salmonella* Gallinarum) causes a severe septicemic disease known as fowl typhoid in broiler chickens and various bird species. Fowl typhoid leads to significant economic losses in commercial poultry production (1). Clinically, *Salmonella* Gallinarum infection presents with diarrhea, dehydration, weakness, and unpredictable morbidity and mortality with lesions developing in multiple organs, and the highest bacterial counts in the liver, spleen, and intestines, especially the caeca (2). The primary treatment for fowl typhoid includes antibiotics such as ciprofloxacin, furazolidone, ampicillin, and gentamycin. However, frequent antibiotic use for both therapeutic and prophylactic purposes has contributed to the emergence of *Salmonella* Gallinarum strains resistant to commonly used antibiotics, including β-lactams, quinolones, fluoroquinolones (e.g., nalidixic acid, ciprofloxacin), and tetracycline (3).

The rise in multidrug-resistant bacteria presents a serious threat to both animal and human health. As a result, there is a growing need for alternative strategies in poultry production, particularly approaches that reduce antibiotic use while promoting gut health and pathogen resistance (4). The increasing resistance to antibiotics, particularly quinolones, underscores the urgent need to find alternative strategies for managing fowl typhoid.

Phytochemicals have gained popularity as potential alternatives to antibiotics due to their antibacterial, antioxidant, and antifungal properties (5). One such plant, *Rauwolfia serpentina* L. Benth. ex Kurz (*Rauwolfia serpentina*), commonly referred to as snakeroot, has traditionally been used to treat various conditions, including hypertension, intestinal diseases, snake bites, breast cancer, and infections caused by Gram-positive bacteria (6). Studies have shown that *Rauwolfia serpentina* root extracts possess antibacterial, antifungal, anti-inflammatory, and antioxidant activities (7), suggesting its potential as a candidate for mitigating the effects of *Salmonella* Gallinarum infection. An *in vivo* study conducted in rats showed that administering 600 mg/kg of *Rauwolfia serpentina* root extract reduced edema, while 800 mg/kg exhibited anti-inflammatory activity anti-bacterial (8), along with antifungal activity and bacteriostatic ability against *Salmonella* typhi (9).

Research findings indicate that reserpine, an alkaloid derived from *Rauwolfia serpentina*, demonstrates significant antimicrobial properties against Salmonella. Experimental studies have revealed enhanced Salmonella elimination in reserpine-treated explants*: In vitro* study using tissue explants from 21 day old broiler chicks showed that when treated with reserpine, these samples exhibited a higher rate of Salmonella destruction compared to untreated controls. This suggests that reserpine may augment the tissue's innate defense mechanisms against this pathogen. *In vivo* studies involving oral reserpine treatment demonstrated a notable decrease in the population of *Salmonella* Typhimurium within the intestinal tract. Moreover, this treatment appeared to have a broader impact, reducing the overall levels of Enterobacteriaceae in the gut (10). These findings underscore reserpine's potential as an antimicrobial agent, particularly in combating Salmonella infections. *Rauwolfia serpentina* has numerous pharmacological properties, including antimicrobial, anti-inflammatory, and stress reliever and immune-modulating effects, which may be beneficial in controlling infections and various conditions (11–13).

The intestinal mucosa plays a crucial role as a barrier against both commensal and pathogenic organisms and provides an innate immune response to invading pathogens (14). Salmonella infections typically occur via the oral route, with bacteria invading the intestines, through Peyer's patches and caecal tonsils (3). The bacteria can persist in the intestines by suppressing the host's inflammatory responses, primarily through T-cell modulation particularly regulatory T cells (Treg). Anti-inflammatory Treg cytokines play important role in maintaining balanced immune response. P20K mRNA expression levels variation were observed in spleen of chickens challenged with *S*. Enteritidis (15). Suppressor of cytokine signaling 3 (SOCS3) protein, a member of SOCS family plays a crucial role in regulating inflammatory and immune responses through multiple mechanisms, negative feedback regulation of the Janus kinase-signal transducer and activator of transcription (JAK/STAT) signaling pathway, which leads to the suppression of immune responses by inhibiting T cell differentiation. Additionally, SOCS3 exerts an anti-inflammatory effect by repressing the M1 proinflammatory macrophage phenotype and deactivating inflammatory responses in macrophages. This dual action on both adaptive and innate immune cells underscores SOCS3's importance in modulating inflammation (16, 17). The major histocompatibility complex (MHC) Class IIβ molecules are crucial for antigen presentation to CD4+ T cells, playing a central role in adaptive immunity. All three genes (SOCS3, P20K, and MHC Class IIβ) were differentially expressed in a microarray experiment and has shown variation in their expression in response to Salmonella challenge (15, 58).

There is limited understanding of the immunomodulatory effects and gene expression modulation by *Rauwolfia serpentina* in broiler chickens challenged with *Salmonella* Gallinarum. While previous studies have highlighted the antimicrobial, antifungal, and anti-inflammatory properties of *Rauwolfia serpentina* and its alkaloid reserpine (7, 9), most of this research has been conducted in non-avian models, such as rats (8), or has focused on different Salmonella serovars like *S*. Typhi (10), rather than *Salmonella* Gallinarum. This study aims to examine the effects of *Rauwolfia serpentina* on growth performance, gene expression of SOCS3, P20K and MHC Class IIβ as well as the immune response in broilers challenged with *Salmonella* Gallinarum. We hypothesized that supplementation with *Rauwolfia serpentina* will modulate gene expression, improve intestinal health and enhance growth performance in broilers infected with Salmonella Gallinarum.

## 2 Materials and methods

### 2.1 Ethical approval

Animal Ethics Committee of the University of Veterinary and Animal Sciences, Lahore, approved all the experimental procedures (Ref. No. DR/350 dated 24-07-2023).

### 2.2 Collection and authentication of *Rauwolfia Serpentina* root material

*Rauwolfia serpentina* root material was purchased from Dr. Masood Pharmaceuticals (Pvt.) Ltd., Lahore, and authenticated by a taxonomist at Government College University, Lahore (specimen voucher: GC.Herb.Bot.2324). The roots were washed, dried and milled into a powder that was used in the birds' diet. Two experimental diets were used one with 15 and 30 g/kg *Rauwolfia serpentina* root powder in the diet. A proximate analysis (18) and phytochemical analysis of *Rauwolfia serpentina* were performed for qualitative and quantitative screening of the plant.

### 2.3 Phytochemical analysis

#### 2.3.1 Qualitative screening

The presence of steroids, alkaloids, glycosides, tannins, flavonoids, terpenoids, and phenols in *Rauwolfia serpentina* root was assessed by qualitative tests following the procedure described by Gul et al. (19) and Lawal et al. (20).

#### 2.3.2 Quantitative analysis

Total flavonoid content (TFC) was quantified using the method described by Chang et al. (21), with quercetin as the standard. Total polyphenol content (TPC) was estimated using the Slinkard and Singleton (22) method, with Gallic acid as the standard. Alkaloid content was assessed using the method outlined by Kokate et al. (23).

### 2.4 Gas chromatography-mass spectrometry (GC-MS) analysis

Gas Chromatography-Mass Spectrometry (GC-MS) analysis was performed using an Agilent Technologies GC-MS system (California, USA) with a 6850 Network GC system and 5973-mass selective detector. Compound identification was done by comparing with the NIST 05 Mass Spectral Library and published spectra (24).

### 2.5 Experimental design and animal husbandry

A total of 240 Hubbard Classic chickens were randomly assigned to four experimental groups, each consisting of four replicates with 15 birds per replicate. The treatment groups included: non-challenge control (NC), *Salmonella* Gallinarum challenge only (SGC), 1.5% *Rauwolfia serpentina* powder + challenge (RSP-1), and 3% *Rauwolfia serpentina* powder + challenge (RSP-2). Bacteriological analysis of fecal samples and serum plate agglutination test using *Salmonella* Gallinarum antigen was performed to make sure Salmonella-free status of chickens (25). On day 7, all the birds in the SGC group, RSP-1 and RSP-2 received the *Salmonella* Gallinarum challenge. In addition, challenged birds in RSP-1 and RSP-2 groups received a diet supplemented with 1.5% (15 g/kg) and 3% (30 g/kg) of *Rauwolfia serpentina* root powder respectively from day 3 till day 28 of age. During the experiment, all birds were housed in floor pens within climate-controlled rooms. The temperature was maintained between 33 and 30°C during the first 14 days, after which it was gradually reduced to 21°C by day 28. Throughout the study, all birds had unrestricted access to feed and water.

### 2.6 *Salmonella* Gallinarum challenge

*Salmonella* Gallinarum stock, previously used in Liaqat et al. (26), was thawed and cultured on Salmonella Shigella Agar (Oxoid, UK) and Brilliant Green Agar (Oxoid, UK). An individual bacterial colony was transferred into Brain Heart Infusion Broth (Oxoid, UK) and allowed to grow overnight in an incubator maintained at 37°C. Birds in the challenge groups received 1 ml of BHI broth containing 2 × 108 CFU of *Salmonella* Gallinarum via oral gavage on day 7, while control birds received an equivalent volume of sterile BHI broth. Following the challenge, chickens were monitored twice daily for signs and symptoms of fowl typhoid, as described by Saleem et al. (2) till the end of the experiment.

### 2.7 Sample collection and processing

On days 3 and 21 post-challenge, five birds from each replicate were humanely euthanized by cervical dislocation for post-mortem examination. Following necropsy, tissue samples from the caeca, spleen and liver were aseptically collected to evaluate expression levels of SOCS3, P20K and MHC CLASSIIβ genes that represent classical immune response, and stored in RNAlater^®^ solution (Thermo Fisher Scientific, Waltham, MA, USA) at −80°C until further processing.

### 2.8 RNA extraction and gene expression analysis

Total RNA was extracted using TRIzol^®^ Reagent (27) according to the manufacturer's recommendation. Quantity and quality of RNA were assessed using a NanoDrop™ 2000 spectrophotometer and Bioanalyzer (Agilent 2100). Samples with an RNA Integrity Number (RIN) above 8.0 were utilized for subsequent analyses.

For the synthesis of complementary DNA (cDNA), 1 μg of RNA was reverse-transcribed using the High-Capacity cDNA Reverse Transcription Kit (Thermo Fisher Scientific). Quantitative real-time PCR (qPCR) was conducted in technical triplicate with SYBR Green chemistry using a Thermal Cycler (Applied Biosystems™ Veriti). The reactions were carried out in a total volume of 20 μl, containing 10 μl of 2 × SYBR Green Master Mix (Thermo Fisher Scientific), 0.5 μl of each forward and reverse primer (10 μM), 2 μl of cDNA template, and 7 μl of nuclease-free water. GAPDH was used as the endogenous control. The thermal cycling conditions included an initial denaturation at 95°C for 10 min, followed by 45 cycles of a three-step touchdown protocol (15). Melt curve analysis was performed to confirm amplification specificity. Gene expression levels for SOCS3, P20K, and MHC CLASSIIβ were quantified using previously validated primers (15). Primer sequences and gene accession numbers are provided (in Supplementary Table 1). Relative gene expression was determined using the 2^−ΔΔCt^ formula (28).

### 2.9 Histopathological and morphometric analysis

Caecal tissue samples from five birds from each replicate were collected on day 21 post-infection (in addition to gene analysis), washed with sterile saline solution to remove the digesta immersed in fixative (10% buffered formalin) followed by embedding in paraffin and cutting into 4 μm sections (29) and stained with H&E and Periodic Acid–Schiff (PAS). The slides were analyzed using a light microscope (Olympus CX31) with a digital imaging system (Olympus DP20, Olympus USA). The morphometric analysis included measurements of villus height (VH), crypt depth (CD), VH and CD ratio, and surface area calculated using the formula (2π) (VW/2) (VL). Goblet cell numbers, acidic and neutral mucin were also assessed (30).

### 2.10 Growth performance

Body weights (BW) were recorded for each replicate at day 0, 7 and 28 days of age. Body weight gain was calculated using the initial BW at the beginning and on days 7 and 28 days of age. Feed intake was also recorded at day 7 and 28 days of age. FCR were calculated to assess the growth performance of birds.

### 2.11 Statistical analysis

Data were analyzed using Genstat 11 for Windows (VSN International Ltd, Hemel Hempstead, UK). For qPCR data, relative gene expression was calculated using the 2^−ΔΔCt^ method. The pen served as the experimental unit for growth performance data, while the individual bird was the unit for histopathological, morphometric, and gene expression analyses. Morphometric parameters, including villus height (VH), crypt depth (CD), VH:CD ratio, surface area, and goblet cell counts, as well as mucin composition (acidic and neutral), were analyzed using analysis of variance (ANOVA) to evaluate the effects of treatments on caecal histomorphometry and mucin production. No outliers were removed prior to analysis. Gene expression data were then analyzed using a one-way analysis of variance (ANOVA). *Post-hoc* comparisons were conducted using Bonferroni's test to determine significant differences between specific groups. Differences were considered statistically significant at *p* < 0.05. Results are presented as means ± standard error of the mean (SEM).

## 3 Results

The proximate analysis of *Rauwolfia serpentina* root showed the sample contained 5% moisture, 7% total ash, 4% water-soluble ash, 2% acid-insoluble ash, and 1% sulphated ash. Alcoholic and aqueous extractions yielded 22% and 34%, respectively. Qualitative and quantitative analysis of the crude powder of *Rauwolfia serpentina* root revealed the phytochemical composition (Tables 1, 2). Preliminary phytochemical analysis showed the presence of alkaloids, tannin and glycosides. Quantitative analysis indicated that alkaloids were present in significantly higher concentrations compared to polyphenols and flavonoids (Table 2). The GC-MS analysis identified various compound of *Rauwolfia serpentina* root powder and their respective immunomodulatory and biological activities, such as Reserpine, 7-Hydroxycoumarin, and Oleic acid. Each compound is referenced with supporting literature, highlighting their diverse therapeutic potentials (Table 3).

**Table 1:** Phytochemical composition of crude powder of *Rauwolfia serpentina* root powder.

**Parameters**	***Rauwolfia serpentina* (Crude powder)**
Alkaloid	+
Tannin	+
Steroid	+
Cardiac glycoside	–
Saponin	+
Flavonoid	+
Terpenoid	+

(+) constituent present, (–) constituent absent.

**Table 2:** Quantitative phytochemical analysis of crude powder of *Rauwolfia serpentina* root powder.

**Total flavonoid content, TFC (QE mg/g)**	**Total polyphenol content, TPC (GAE mg/g)**	**Total alkaloids (%)**
4.3 ± 0.3	2.7 ± 0.3	7.4 ± 0.4

**Table 3:** Identified compounds in GC-MS analysis in *Rauwolfia serpentina* root powder with immunomodulatory activity.

**Sr. No**	**Compound**	**Activity**
1.	Reserpine	Immune system stimulator (40)
2.	2-pentanol,acetate	• Antibacterial (44) • Anticancer (45)
3.	2 Methoxy-4-vinyl phenol	Antibacterial (46), anti-inflammatory, analgesic, antioxidant (47)
4.	2,4-di-tert-butylphenol	Antibacterial, inhibitory effect on plant immune system and seed germination, potent toxicity
5.	Naphthalene	Stimulatory and Inhibitory effects upon cell proliferation (48)
6.	7-Hydroxycoumarin	Protective immune responses (49)
7.	Oleic acid	Elevated the immune responses of hybrid groupers toward Vibrio infection (50)
8.	Farnesiferol A, acetate	Anti-tumor, immune modulator (34)
9.	Trismethoxyresveratrol, trans–	Anti-bacterial (51), anti-inflammatory, anti-oxidant (52), Immune system modulation to potentiate anti-tumor immunity (53)
10.	Bis (2-ethylhexyl) phthalate	Adaptive immune system as a promoter of Th1/Th2 immune responses (54), promotes tumor immune escape and activates signaling pathways (55)
11.	Trans-4-Fluoro-4-methoxychalcone	Antibacterial (56)
12.	3-Hydroxy-4,5-dimethoxystilbene	Antibacterial, anti-inflammatory, immune regulation (57)

### 3.1 Clinical observations

**Figure 1:**
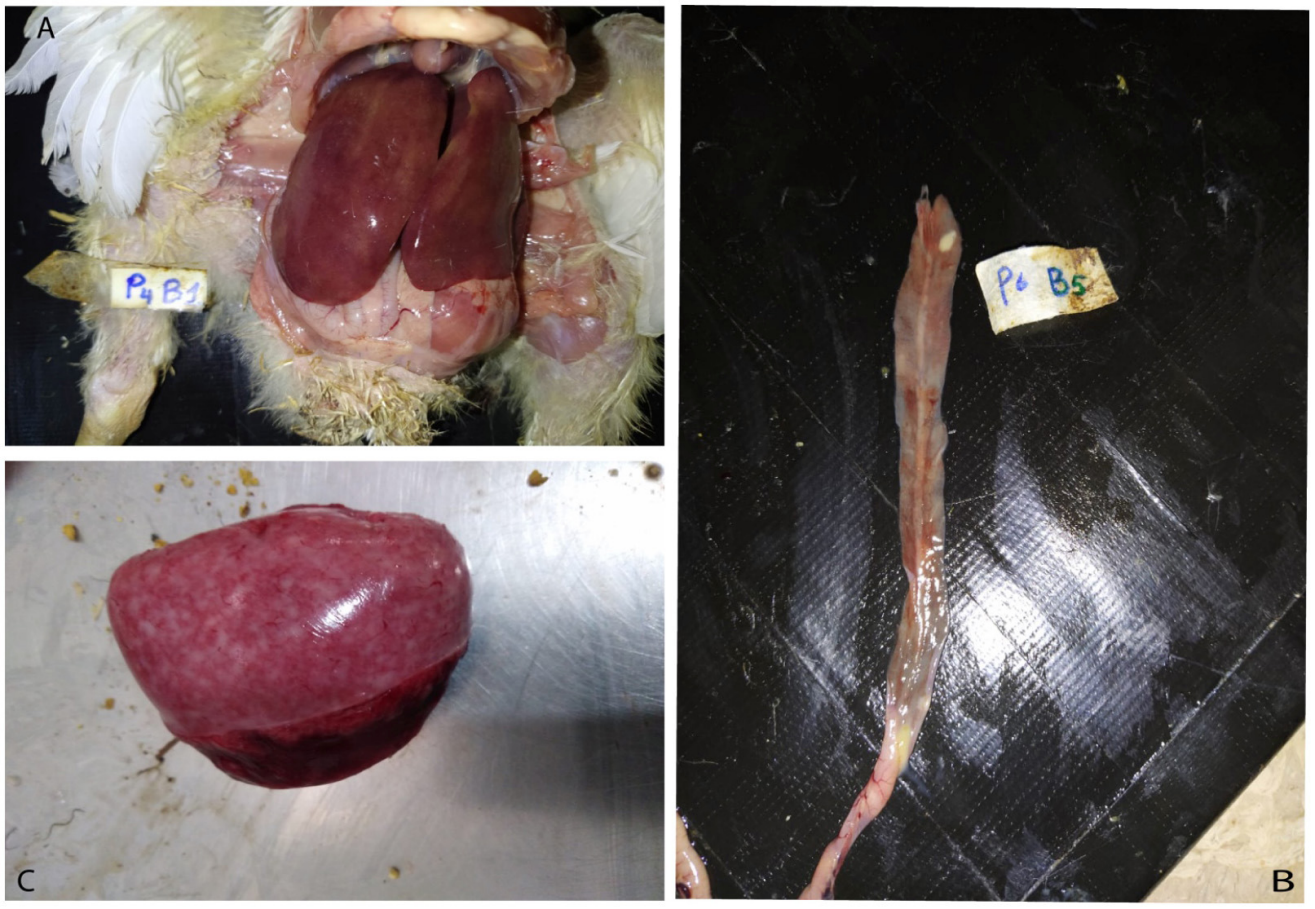
Gross lesions in broiler chicks experimentally infected with *Salmonella* Gallinarum. **(A)** Enlarged and hemorrhagic liver with characteristic swelling and bronze discoloration. **(B)** Enteritis with areas of necrosis. **(C)** Splenomegaly.

During daily clinical observations, ~15% of the birds in the SGC group exhibited symptoms consistent with fowl typhoid, including ruffled feathers, rapid respiration, emaciation, and yellowish-white diarrhea. During the experiment, 1% of the chickens died. None of these deaths showed evidence of being caused by the *Salmonella* Gallinarum infection. On day 10 (3 days post-challenge), a post-mortem examination revealed moderate liver discolouration with hemorrhages and caeca lesions, predominantly inflammation, along with splenomegaly in the SGC group (Figure 1). This indicated a moderate severity of the challenge. In contrast, birds supplemented with *Rauwolfia serpentina* at 1.5% and 3%, exhibited milder effects with only three out of 10 and two out of 10 birds, respectively, showing mild liver discolouration with necrotic foci. Additionally, only a very mild degree of enteritis was observed in these birds.

### 3.2 Gene expression analysis

**Figure 2:**
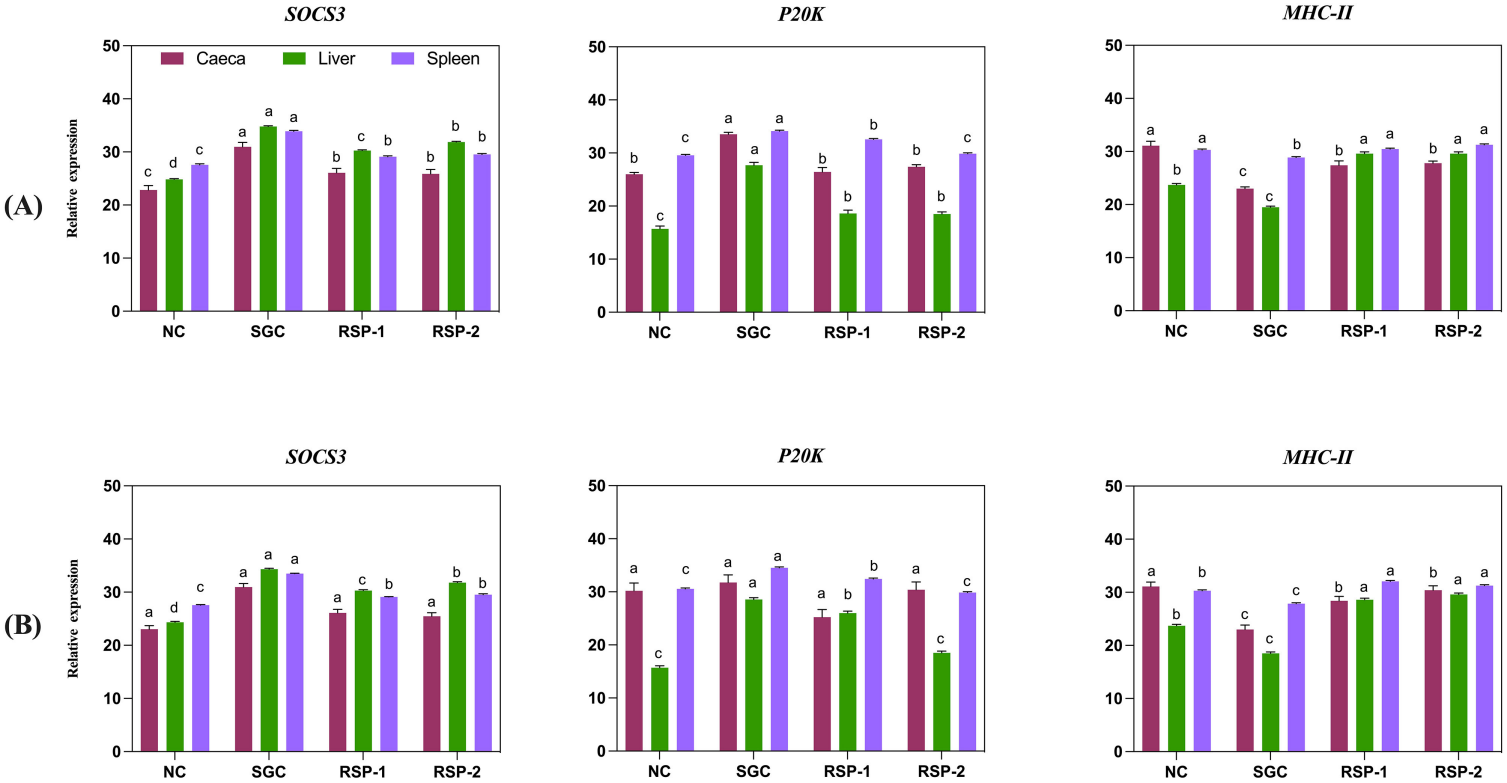
Effect of *Rauwolfia serpentina* root supplementation on expression levels of SOCS3, P20K and MHC class IIβ gene in caecum, liver and spleen of broiler chicks experimentally challenged with *Salmonella* Gallinarum **(A)** 3 days post-challenge **(B)** 21 days post-challenge. (NC) Non-challenge control group (SGC), *Salmonella* Gallinarum challenge group (RSP-1) group fed basic diet + 1.5% *Rauwolfia serpentina* root along with *Salmonella* Gallinarum challenge (RSP-2) group fed basic diet + 3% *Rauwolfia serpentina* root + *Salmonella* Gallinarum challenge. Values are represented as means and SEM are represented by vertical bars and different letters (a-b-c-d) indicate a significant difference.

The expression levels of three key immune-related genes-SOCS3, P20K, and MHC class IIβ were analyzed in the caecum, liver, and spleen of broiler chicks at 3 and 21 days post-challenge against *Salmonella* Gallinarum. The effects of *Rauwolfia serpentina* supplementation were compared to both NC and SGC (Figure 2).

On day 3 post-challenge, SOCS3 gene expression varied significantly across treatments and tissue (caeca, liver, and spleen: *p* < 0.05). The SGC birds exhibited the highest SOCS3 expression levels in the caecum, liver, and spleen, while birds supplemented with RSP-1 and RSP-2 showed significantly lower expression levels than the SGC group (Figure 2A). In the spleen, SOCS3 expression in RSP-1 and RSP-2 groups was still significantly higher than in the NC group (*p* < 0.05).

Supplementation of *Rauwolfia serpentina* had a significant effect on SOCS3 gene expression in the liver and spleen (*p* < 0.001) on day 21 post-challenge, while no significant differences (*p* = 0.061). were observed in the caeca (Figure 2B). SGC birds exhibited the highest SOCS3 expression in both the liver and spleen, with significantly lower expression observed in birds supplemented with *Rauwolfia serpentina* (RSP-1 and RSP-2) compared to the SGC group. However, SOCS3 levels in the supplemented groups remained higher than in the unchallenged birds. Both supplementation levels (1.5 and 3%) resulted in similar SOCS3 expression in the spleen, significantly lower than the SGC group but higher than the unchallenged birds (Figure 2B).

On day 3 post-challenge, P20K gene expression showed tissue-specific variations across treatment groups (Figure 2A). In all three organs, both RSP-1 and RSP-2 groups exhibited significantly lower P20K expression than SGC groups (*p* < 0.05). Expression of P20K was highest in the SGC group, significantly higher than all other groups (*p* < 0.05). In the liver, supplementation of *Rauwolfia serpentina* resulted in intermediate expression levels, significantly higher than NC but lower than SGC (*p* < 0.05). In the spleen, P20K expression was significantly elevated in all SGC group compared to the other treatment group (*p* < 0.05). At 21 days post-challenge, caecal P20K expression showed no statistically significant differences among groups (*p* = 0.111, Figure 2B).

MHC class IIβ gene expression patterns varied across tissues and treatment groups on day 3 post challenge (Figure 2A). SGC groups showed significantly lowest expression in all three tissues examined compared to all other treatment groups (*p* < 0.05). Liver MHC class IIβ expression was significantly elevated in all treatment groups compared to the NC group (*p* < 0.05). The SGC group showed the lowest expression, followed by RSP-1 and RSP-2, all significantly different from the NC group (*p* < 0.05). On day 21 post-challenge, significant differences were observed in MHC Class IIβ expression across all tissues (caeca: *p* = 0.008, liver and spleen: *p* < 0.001). SGC birds exhibited the lowest expression levels in the caecum, liver, and spleen, whereas birds supplemented with RSP-2 and RSP-1 showed significantly higher expression levels than SGC group (Figure 2B).

### 3.3 Histopathological and morphometrical analysis of caeca

**Figure 3:**
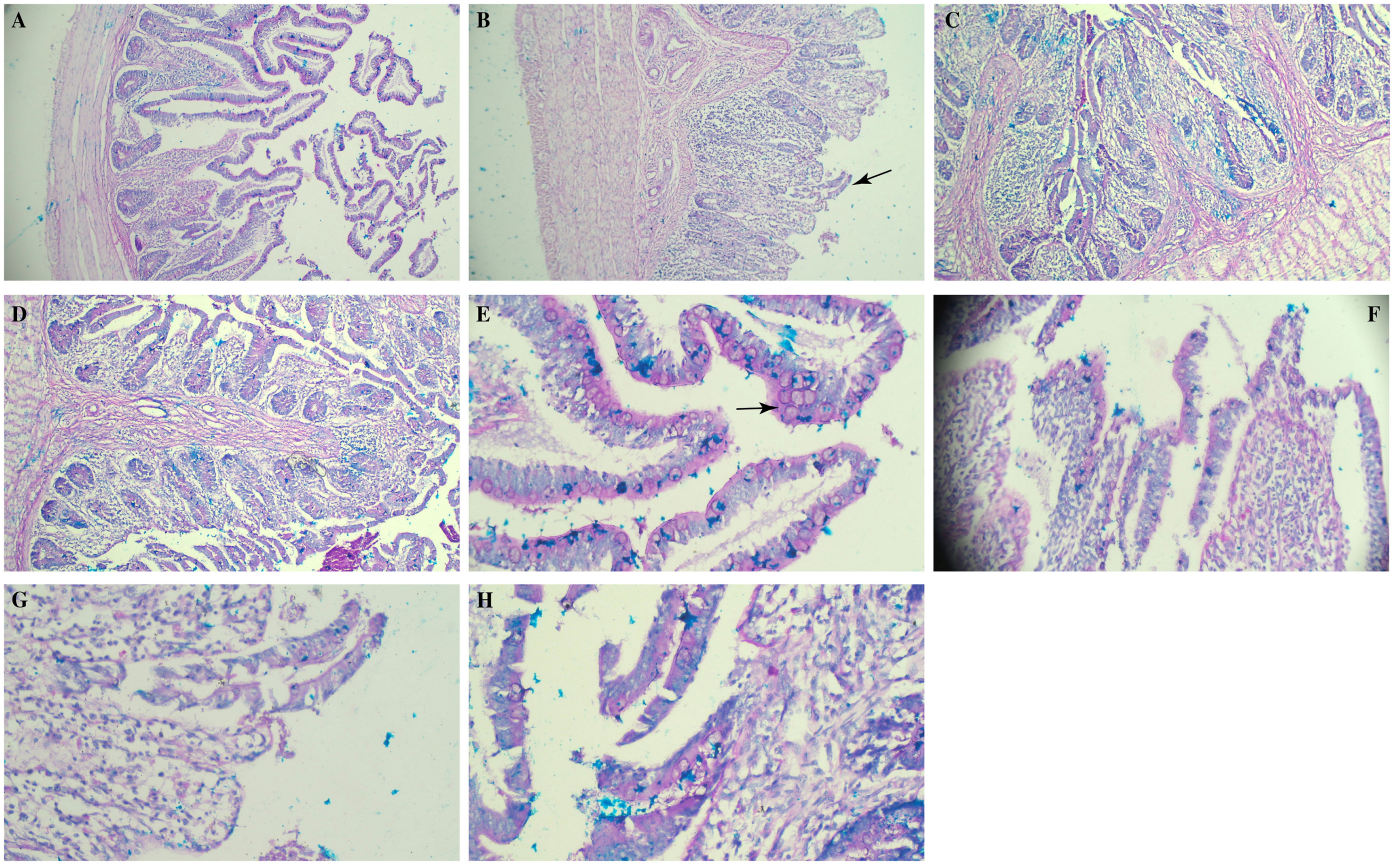
Photomicrograph showing the effect of *Rauwolfia serpentina* root supplementation on caeca of broiler chicks experimentally challenged with *Salmonella* Gallinarum (21 days post-challenge). **(A, E)** Non-challenge control group, **(B, F)** *Salmonella* Gallinarum challenge group, **(C, G)** group fed a basic diet + 1.5% *Rauwolfia serpentina* root along with challenge, **(D, H)** group fed a basic diet + 3% *Rauwolfia serpentina* root + *Salmonella* Gallinarum challenge. **(B)** Arrow shows ruptured villi **(E)** arrow shows goblet cells in the villous (PAS), separation in the epithelium of villous. Upper row—*10*×*;* lower row–40×.

Histopathological examination of caecal tissue revealed marked disruption of normal architecture and abnormal mucin production in SGC birds (Figure 3B). Alcian Blue staining showed intense mucin production, accompanied by areas of necrosis, tissue degeneration and inflammatory infiltrates (Figure 3F). These findings suggest a progressive pathological process affecting the tissue, likely impairing nutrient absorption and mucosal defense. In contrast, birds supplemented with RSP-2 and RSP-1 showed only light to moderate changes, with mucin production and tissue architecture largely intact. Cellular morphology appeared mostly regular, with only slight nuclear size and shape variations, suggesting that *Rauwolfia serpentina* supplementation contributed to tissue repair.

Morphometric analysis indicated that *Rauwolfia serpentina* supplementation had a significant impact on caecal morphology (Table 4). Villus height (VH) was significantly greater in the RSP-2 (707 μm) and RSP-1 (706 μm) groups compared to the SGC group (267 μm; *p* = 0.008). Crypt depth was also preserved in the RSP-2 (173 μm) and RSP-1 (167 μm) groups, significantly higher than in the SGC group (74.3 μm; *p* = 0.002). Although the VH ratio did not differ significantly among the groups (*p* = 0.675), the surface area of the caecum was significantly larger in the RSP-2 (738 μm) and RSP-1 (743 μm) groups compared to the SGC group (145 μm; *p* = 0.005). The number of goblet cells in the RSP-2 group was significantly higher (*p* = 0.01), with an average of 12 cells per section, compared to five cells in the SGC group. Acidic and neutral mucin levels did not differ significantly between treatments (*p* = 0.079 and *p* = 0.222, respectively).

**Table 4:** Effect of *Rauwolfia serpentina* root powder supplementation on morphometric analysis of caecum of broiler chicks experimentally challenged with *Salmonella* Gallinarum (21 days post-challenge).

**Group**	**VH (μm)**	**CD (μm)**	**VH:CD**	**Surface area (μm)**	**Goblet cells**	**Acidic mucin**	**Neutral mucin**
NC	676^b^	172^a^	3.93	703^a^	8.33^ab^	5	3.33
SGC	267^c^	74.3^b^	3.73	145^b^	5^b^	2.67	2.33
RSP-1	706^a^	167^a^	4.42	743^a^	9.33^ab^	6.33	3
RSP-2	707^a^	173^a^	4.07	738^a^	12^a^	6.67	5.33
*p*-value	0.008	0.002	0.675	0.005	0.01	0.079	0.222
SEM	77.9	13.67	0.4	95.3	1.067	1	0.957

NC, non-challenge control group; SGC, *Salmonella* Gallinarum challenge group; RSP-1, group fed basic diet + 1.5% *Rauwolfia serpentina* root + *Salmonella* Gallinarum challenge; RSP-2, group fed basic diet + 3% *Rauwolfia serpentina* root with *Salmonella* Gallinarum challenge; VH, villi height; CD, crypt depth; VH:CD, villus height and crypt depth ratio; SEM, standard error of means.

Values are represented as means and SEM.

^a, b, c^Means in the same column with different superscripts are significant at p < 0.05.

### 3.4 Growth performance

During the first week of age, no significant differences in weight gain, FCR, or feed intake were observed among the treatment groups. However, by day 28 (21 days post-challenge), *Rauwolfia serpentina* supplementation had marked effects on growth performance (Table 5). Birds in the RSP-2 group showed significantly higher weight gain (1,687 g) compared to other groups received SGC (*p* = 0.006) except NC group. The SGC group showed lower overall weight gain (1,455 g) compared to all other treatment groups (*p* = 0.001). The RSP-2 group also had significantly lower feed intake (2,379 g) than the other groups received SGC (*p* = 0.003), indicating better feed efficiency. In contrast, feed intake in the RSP-1 group (2,456 g) was comparable to the control groups. These results suggest that *Rauwolfia serpentina* supplementation at both levels offered protective effects against SGC, with the effects becoming more pronounced over time.

**Table 5:** Effect of *Rauwolfia serpentina* root powder supplementation on weight gain, FCR and feed intake of broiler chicks experimentally challenged with *Salmonella* Gallinarum.

**Group**	**Weight gain (g)**		**FCR**		**Feed intake (g)**	
	**Day 7**	**Day 28**	**Day 7**	**Day 28**	**Day 7**	**Day 28**
NC	127.5	1,723^a^	1.5	1.660^b^	189	2,294^bc^
SGC	131	1,455^c^	1.4	1.850^a^	187	2,693^a^
RSP-1	129.3	1,628^b^	1.50	1.515^bc^	191	2,456^b^
RSP-2	124.5	1,687^ab^	1.55	1.475^c^	189	2,379^c^
*p*-value	0.372	0.006	0.068	0.001	0.093	0.003
SEM	2.40	27.3	0.0212	0.023	0.791	43.9

NC, non-challenge control group; SGC, *Salmonella* Gallinarum challenge group; RSP-1, group fed basic diet + 1.5% *Rauwolfia serpentina* root + *Salmonella* Gallinarum challenge; RSP-2, group fed basic diet + 3% *Rauwolfia serpentina* root with *Salmonella* Gallinarum challenge.

Values are represented as means and SEM.

^a, b, c^Means in the same column with different superscripts are significant.

## 4 Discussion

The GC-MS analysis of the methanol extract of *Rauwolfia serpentina* identified 30 distinct compounds based on their retention time, percentage area, molecular weight, and molecular formula (Supplementary Table 2). Among these, 12 compounds have been reported in the literature to exhibit various biological activities, including antibacterial, anticancerous, anti-inflammatory, and antioxidant properties (Table 3). Notably, compounds such as 2,4-di-tert-butylphenol, naphthalene, 7-hydroxycoumarin, oleic acid, farnesiferol A acetate, trismethoxyresveratrol (trans-), bis (2-ethylhexyl) phthalate, and 3-hydroxy-4,5-dimethoxystilbene, reserpine have been shown to influence the immune system (Table 3).

### 4.1 Gene expression analysis

Present study revealed significant variation in the expression of three key immune-related genes- SOCS3, P20K, and MHC class IIβ in broiler chicks supplemented with *Rauwolfia serpentina* and challenged with *Salmonella* Gallinarum. Supplementation with *Rauwolfia serpentina*, significantly modulated the tissue specific expression of these genes in response to *Salmonella* Gallinarum challenge.

In particular, gene expression patterns varied across the caecum, liver, and spleen. SOCS3 was significantly upregulated in the SGC group, followed by P20K, while MHC class IIβ expression was lowest in these tissues. The differential expression of immune-related genes in these organs following pathogen exposure underscores the importance of a coordinated immune response during the early stages of systemic infection (15). Following the invasion, Salmonella activates the host immune responses by invading macrophages and subsequent activation of pro-inflammatory cytokines such as IL-6 and TNF-α, responsible for the bird's innate and adaptive immune responses.

Suppressor of cytokine signaling 3 (SOCS3) plays a crucial role in regulating TNF-α, IL-6, and IL-12 (31, 32). In mouse dendritic cells, SOCS3 has been found to inhibit IL-12 expression, which can shift the immune response toward a Th2 profile along with decreased expression of MHC class IIβ (33). This explains the down-regulation of MHC class IIβ expression observed in SGC chicks. MHC class II, consisting of alpha and beta chains, is predominantly expressed on antigen-presenting cells such as macrophages, dendritic cells, and phagocytes. The variability of avian MHC haplotypes influences host susceptibility or resistance to various pathogens (34). In the current study, significant upregulation of SOCS3 in the caecum, liver, and spleen of birds challenged with *Salmonella* Gallinarum likely indicates an attempt to moderate the inflammatory response against infection (35). As the immune response of birds to Salmonella infection primarily involves a pro-inflammatory reaction, Salmonella is known to evade host immunity by upregulating the expression of SOCS3, thereby inhibiting the JAK/STAT pathway, leading to reduced production of cytokines like IFN-γ responsible for bacterial clearance and inhibition of MHC class IIβ expression to avoid detection of the immune response and persist in infection (36, 59).

A balanced adaptive immune response relies on the proper coordination and regulation of Th1 and Th2 cell activation, facilitated by Treg cells (37). The quiescence-related gene P20K (P20K) was significantly elevated in the liver and spleen of *Rauwolfia serpentina*-supplemented birds. This upregulation might indicate a Treg response, crucial in balancing the immune response to *Salmonella* Gallinarum. This differential expression of P20K across different tissues suggests that *Rauwolfia serpentina* might modulate host responses in a tissue-specific manner. This is particularly evident by an exhibition of different patterns of MHC Class IIβ expression across caeca, liver and spleen on days 3 and 21 post-challenge. MHC Class IIβ gene has a crucial role in initiating adaptive immune responses by presenting antigens to CD4+ T cells. On day 21 post-challenge, SGC birds exhibited the lowest expression of MHC class IIβ levels, whereas birds supplemented with *Rauwolfia serpentina* showed significantly higher expression levels indicating that *Rauwolfia serpentina* supplementation upregulated MHC class IIβ expression, in the spleen and caeca. This early immune response in current study is likely due to the initial activation of the immune system in response to both the dietary supplementation and *Salmonella* Gallinarum challenge. The downregulation of MHC class IIβ expression in the caecum of SGC birds aligns with previous findings (15), suggesting that *Salmonella* Gallinarum may evade host defense by suppressing MHC class IIβ expressions. Studies have shown that *Salmonella* Enteritidis can evade the Th1 immune response by inducing SOCS3 up-regulation, which in turn inhibits IFN-γ, leading to reduced MHC class IIβ expression (15, 38, 39). In contrast, the upregulated MHC class IIβ expression in the *Rauwolfia serpentina-*supplemented groups suggests that phytochemicals in the plant may promote persistent antigen presentation and enhance immune responses, even in the absence of active infection. This may be attributed to immune-boosting and anti-inflammatory properties of *Rauwolfia serpentina* (40). This tissue-specific difference observed in this study highlights the complexity of the immune responses to systemic pathogen challenges and aligns with previous studies of variable immune responses between systemic and localized immune systems in broiler chicks challenged with *Salmonella* Gallinarum (15). The immunomodulatory effects of Rauwolfia *serpentina* against *Salmonella* Gallinarum observed in the current study may be attributed to its potential chemical compounds, reserpine, saponins, tannins, flavonoids, and terpenoids. It particularly affects the release of norepinephrine with subsequent elevation of cytokine production and cell proliferation. We hypothesized that reserpine, along with other potential compounds present in *Rauwolfia serpentina*, may interplay host immune system, therefore activating several metabolic pathways. These pathways likely to enhance the production of antimicrobial peptides, enhanced IL-2 expression with reduced CTLA-4 expression therefore promoting T cell activation and proliferation in chickens (41). Additionally, reserpine has been shown to improve gut health by influencing antimicrobial pathways, mainly epidermal growth factor receptor (EGFR) and mammalian target of rapamycin (mTOR) and elevating AMP gene expression while decreasing the number of Enterobacteriaceae and Salmonella counts (10).

### 4.2 Intestinal morphology and gut health

The results of the current study revealed mucosal damage, as indicated by a reduced VH and CD in broiler birds challenged with *Salmonella* Gallinarum. However, supplementation with *Rauwolfia serpentina*, especially in the RSP-2 group, resulted to an increase in goblet cell numbers in the caeca, suggesting improved gut health. Goblet cells are essential for mucus secretion, forming a protective barrier against pathogens and facilitating nutrient absorption (42). The current study further revealed a significant suppressive effect of the Salmonella challenge on goblet cell numbers, as indicated by a notable decrease in goblet cell numbers in the caeca of SGC broiler chickens. This finding aligns with observations of gut morphology, where a reduced VH to CD ratio was evident in broiler chicks that received *Salmonella* Gallinarum challenge. Prior research has similarly demonstrated a significant decrease in VH and CD in the intestines of *Salmonella* Typhimurium-infected birds, indicating potential mucosal damage caused by this pathogen.

Intestinal morphology, measured through duodenal and ileal VH and CD, as well as the VH to CD ratio, serves as an indicator of gut health in broilers (42). In addition to increase in goblet cells as evident by histomorphology (Figure 3D) coupled with improved villus morphology (Figure 3; Table 4), mainly increase in surface area that helps in better nutrient absorption, indicating a larger surface area for enzymatic activity and nutrient uptake (43) suggests that *Rauwolfia serpentina* root may enhance the intestinal barrier function and overall gut health in broiler chicks against *Salmonella* Gallinarum.

### 4.3. Growth performance

In the present study, growth performance parameters further support the potential benefits of *Rauwolfia serpentina* supplementation against *Salmonella* Gallinarum. During the first week of the trial, no significant differences were observed in feed intake, weight gain, or FCR across different treatment groups. However, by the fourth week, the RSP-2 birds demonstrated the best FCR (lower values) among all treatment groups, suggesting a more efficient conversion of feed into body mass. The enhanced growth performance in the RSP-2 group could be attributed to improved gut health, as evidenced by increased VH and goblet cell numbers, potentially leading to better nutrient absorption. Another possible explanation for better FCR could be attributed to a well-regulated immune response, as indicated by the upregulation of key immune-related genes, allowing birds to combat *Salmonella* Gallinarum challenge more effectively without excessively diverting energy from growth. The potential effects of *Rauwolfia serpentina* phytochemicals include promoting beneficial gut microbiota that contributes to improved nutrient utilization (41).

The observation that the birds in RSP-2 group showed slightly better performance in terms of better weight gain and FCR than RSP-1 could be due to the presence of additional beneficial bioactive compounds in the whole root powder or more release of active ingredients and more prolonged action of active compounds in the gut, promoting more sustained immune modulation and better growth performance. The finding that RSP supplementation results in better weight gain and FCR compared to *Salmonella* Gallinarum challenge may be due to multiple reasons, such as improved intestinal morphology leading to better nutrient absorption, reduced pathogen load, and lower inflammation, which allows more energy to be directed toward growth rather than immune responses.

Our findings suggest that *Rauwolfia serpentina* supplementation may offer a multifaceted approach to improving the health and productivity of broiler chicks challenged with *Salmonella* Gallinarum. The combination of enhanced immune gene expression, improved gut morphology, and better growth performance presents a promising picture for the use of this phytogenic as a natural growth promoter and immunomodulator.

## 5 Conclusions

The study suggests that supplementation with *Rauwolfia serpentina* has effectively modulated the expression of key immune-related genes, enhanced gut morphology, and improved growth performance in broiler chicks challenged with *Salmonella* Gallinarum. Notably, *Rauwolfia serpentina* supplementation influenced the expression of key immune genes such as SOCS3, P20K, and MHC class IIβ, with differential patterns observed depending on the specific gene and treatment group. These findings highlight its potential as a phytogenic compound capable of modulating immune responses, reducing inflammation, and counteracting the immune evasion strategies employed by *Salmonella* Gallinarum. The varied changes in gene expression underscore the complexity of the immune-modulatory effects and warrant further investigation to elucidate the underlying mechanisms.

The significant increase in goblet cell numbers and improved villus morphology observed in the *Rauwolfia serpentina*-supplemented groups underscores the plant's positive effects on gut health, which may contribute to enhanced nutrient absorption and overall better growth performance. The findings suggest that the immune-modulatory and antimicrobial properties of *Rauwolfia serpentina*, particularly through compounds like reserpine, could play a crucial role in promoting beneficial gut microbiota and improving feed conversion ratios.

These results contribute to the growing body of evidence supporting the use of phytogenic compounds as immune booster in poultry production. Unlike previous studies that focused primarily on antimicrobial properties, our study is among the first to demonstrate *Rauwolfia serpentina*'s tissue-specific modulation of key immune genes in broilers challenged with *Salmonella* Gallinarum. While our study provides valuable insights, several limitations should be addressed in future research: the study duration was relatively short. Long-term studies could provide insights into the sustained effects of *Rauwolfia serpentina* supplementation on bird health and performance. We focused on a limited number of immune-related genes. A more comprehensive gene expression analysis, possibly using RNA-seq, could provide a broader understanding of the immunomodulatory effects of *Rauwolfia serpentina*.

While these findings are promising, future research should address the limitations of the current study, including the relatively short duration and the focus on a limited number of immune-related genes. Investigating the expression of pro-inflammatory and anti-inflammatory cytokine genes could be particularly valuable in elucidating the balance of immune responses mediated by *Rauwolfia serpentina* supplementation. Long-term studies and comprehensive gene expression analyses could provide deeper insights into the sustained effects and broader immunomodulatory mechanisms of this phytogenic compound.

## Data Availability

The datasets presented in this study can be found in online repositories. The names of the repository/repositories and accession number(s) can be found in the article/Supplementary material.
